# Nurses and Trade Unions

**Published:** 1921-02-19

**Authors:** 


*To the Editor of* The Hospital.

### Nurses and Trade Unions.

*To the Editor of* The Hospital.

Sir,—Sister Caroline writes to you, "Is it not true that Lord Knutsford says that the College can do nothing?" No, it is not true. The only time I have spoken I have said that the College could do a great deal, but could not do everything at once, and I pleaded for patience. The memorandum the College has just issued as to nurses' salaries ought to help. It has been sent to all hospitals.—Yours truly,

Knutsford.

London Hospital, Whitechapel, E.
February 15, 1921.

